# Bibliometric and visualized analysis of 3D printing bioink in bone tissue engineering

**DOI:** 10.3389/fbioe.2023.1232427

**Published:** 2023-07-20

**Authors:** Kaihao Xu, Sanyang Yu, Zhenhua Wang, Zhichang Zhang, Zhongti Zhang

**Affiliations:** ^1^ The VIP Department, School and Hospital of Stomatology, China Medical University, Shenyang, China; ^2^ Department of Physiology, School of Life Sciences, China Medical University, Shenyang, China; ^3^ Department of Computer, School of Intelligent Medicine, China Medical University, Shenyang, China

**Keywords:** bioink, hydrogel, 3D printing, bone tissue engineering, biomaterial, bibliometrics, data visualization

## Abstract

**Background:** Applying 3D printed bioink to bone tissue engineering is an emerging technology for restoring bone tissue defects. This study aims to evaluate the application of 3D printing bioink in bone tissue engineering from 2010 to 2022 through bibliometric analysis, and to predict the hotspots and developing trends in this field.

**Methods:** We retrieved publications from Web of Science from 2010 to 2022 on 8 January 2023. We examined the retrieved data using the bibliometrix package in R software, and VOSviewer and CiteSpace were used for visualizing the trends and hotspots of research on 3D printing bioink in bone tissue engineering.

**Results:** We identified 682 articles and review articles in this field from 2010 to 2022. The journal Biomaterials ranked first in the number of articles published in this field. In 2016, an article published by Hölzl, K in the Biofabrication journal ranked first in number of citations. China ranked first in number of articles published and in single country publications (SCP), while America surpassed China to rank first in multiple country publications (MCP). In addition, a collaboration network analysis showed tight collaborations among China, America, South Korea, Netherlands, and other countries, with the top 10 major research affiliations mostly from these countries. The top 10 high-frequency words in this field are consistent with the field’s research hotspots. The evolution trend of the discipline indicates that most citations come from Physics/Materials/Chemistry journals. Factorial analysis plays an intuitive role in determining research hotspots in this sphere. Keyword burst detection shows that chitosan and endothelial cells are emerging research hotspots in this field.

**Conclusion:** This bibliometric study maps out a fundamental knowledge structure including countries, affiliations, authors, journals and keywords in this field of research from 2010 to 2022. This study fills a gap in the field of bibliometrics and provides a comprehensive perspective with broad prospects for this burgeoning research area.

## 1 Introduction

### 1.1 Current status of bone tissue engineering

With the development of biomedicine in recent years, scientists have been exploring the field of bone defect treatments in depth. Increasing numbers of scientists have carried out multi-level research on bone tissue engineering to understand the physiological structure of natural bone (Florencio-Silva et al., 2015; Wubneh et al., 2018; Hart et al., 2020). Autologous bone grafting (Fillingham and Jacobs, 2016; Stanovici et al., 2016) was considered the clinical “gold standard” for treating bone tissue defects in the past (García-Gareta et al., 2015; Schmidt, 2021). Due to limitations of autologous bone transplantation, such as infection, secondary injury and chronic pain (Dimitriou et al., 2011; Wang and Yeung, 2017), bone tissue engineering has been widely studied as an innovative therapy to reduce patient pain, reduce the complexity of doctors’ operation, and accelerate the recovery of bone tissue (Qi et al., 2021). Bone tissue engineering is the process of culturing and growing numerous autologous osteoblasts, bone marrow stromal cells, or chondrocytes *in vitro* before implanting the cells on a biocompatible natural or synthetic cell scaffold. The scaffolds are subsequently progressively broken down and taken up by the extracellular matrix or the human body. These biomaterial scaffolds can give cells a three-dimensional environment in which to live, breathe, exchange gases, eliminate waste, and develop on three-dimensional scaffolds that have already been built. Following the implantation of the cell hybrid material at the site of the bone defect, the transplanted bone cells multiply as the biomaterial eventually degrades to enable the healing of the damaged bone tissue (Chocholata et al., 2019).

Material selection is an important factor in determining the success of bone defect repair. Metal materials were the earliest materials used in bone tissue engineering, and titanium and titanium alloys are still the preferred implant materials for oral implants (Souza et al., 2019; Xie et al., 2020) and orthopaedics (Fokter et al., 2016; Williams et al., 2016). However, metal materials are not degradable and need to be removed in many cases, which not only increases the difficulty of treatment, but also increases the psychological pressure of patients. Bioceramic has been a popular material in recent years, and hydroxyapatite is the most commonly used example (Khan et al., 2021). Hydroxyapatite is an ideal material for bone tissue engineering with outstanding biocompatibilities (Zhou and Lee, 2011). In addition, biodegradable polymers have been considered and used in bone tissue engineering by a growing number of scientists (Aslam Khan et al., 2021; Guo et al., 2021). Commonly studied polymer materials include gelatin (Zhang et al., 2018), chitosan (LogithKumar et al., 2016), hyaluronic acid (Saravanakumar et al., 2022), alginate (Hernández-González et al., 2020), etc. Scaffolds composed of these single biomaterials have their own advantages and disadvantages, leading to the derivation of composite scaffolds composed of two or more biomaterials. The composite scaffold composed of chitosan and gelatin-based electrospun fiber has unique advantages in the construction of scaffolds with high porosity (Venkatesan et al., 2015). The characterization performance of the composite scaffold composed of alginate and hydroxyapatite is better than that of scaffolds composed of single biomaterial in the fields of bone tissue regeneration, treatment of bone defect and drug delivery (Sikkema et al., 2021).

### 1.2 Research status of 3D printing bioink in bone tissue engineering

3D printing can create a structure that closely matches the individual anatomy and physiology of patients with highly personalized design and matching. Therefore, 3D printing has become a major manufacturing technology in the medical field. This technology has broad applications including stomatology, tissue engineering and regenerative medicine (Liaw and Guvendiren, 2017). Bioinks are inks that can be used in 3D printing, and the ideal bioink used in the medical field can not only provide three-dimensional space to support cells, but also participate in creating the microenvironment required for cell survival (Cidonio et al., 2019).

Because 3D printing can control the volume, geometry and internal structure of tissue scaffolds, it is an important technology for bone tissue engineering. 3D bioprinting methods for bone tissue engineering and the invention of biocompatible bioinks have been the main focus of research in this field. They play important roles in homeostasis, supporting a multifaceted balance among cellular function, cellular dynamics, and mechanical integrity (Zhang et al., 2019). Multiple systems containing inkjet 3D printing-based bioprinting (Chou et al., 2013), extrusion-based bioprinting (Placone and Engler, 2018), and laser-based bioprinting (Ashammakhi et al., 2019) have been invented.

### 1.3 Advantages of bibliometric analysis

**FIGURE 1:**
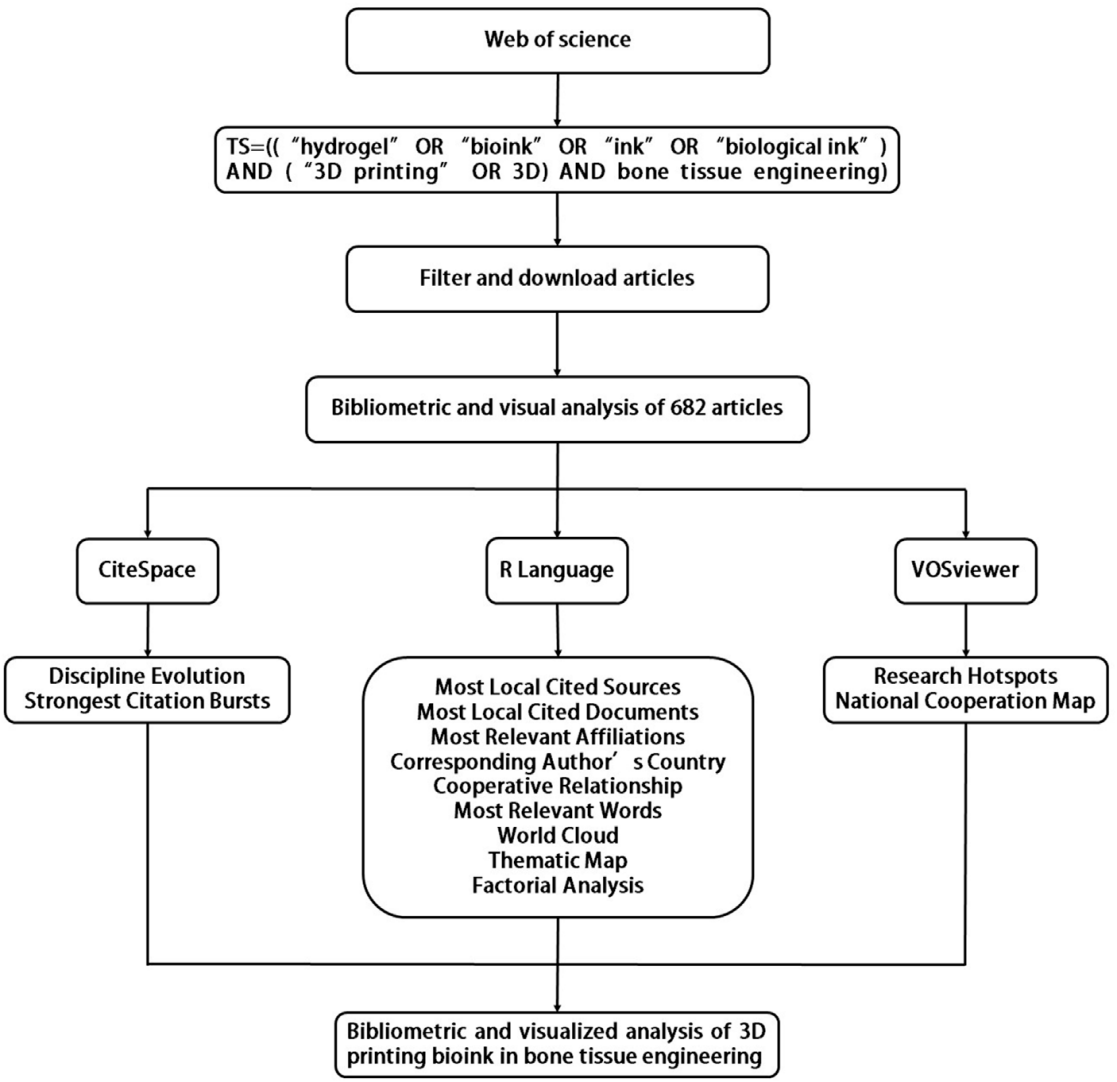
Graphical summary.

Bibliometrics can analyze publications by applying literature details and metrology as objects. Bibliographic systems and bibliometric features are the main research objects of bibliometrics and they adopt quantitative research methods such as mathematics and statistics while studying the distribution structure, quantitative relationship, changing law and quantitative management of bibliographic information (Ge Y. et al., 2022; Li et al., 2022). During the analysis process, relevant information including authors, keywords, journals, countries, references, and cooperation maps are harvested from related research fields (Roldan-Valadez et al., 2019). The development of bibliometric tools such as the R package “Bibliometrix” (Arruda et al., 2022) and the software packages “CiteSpace” (Liu et al., 2022) and “VOSviewer” (Huang et al., 2022) makes visual analysis of documents more convenient. These programs have been widely used in various fields, such as medicine (Youn et al., 2022), climate (Han et al., 2022), materials science (Sun et al., 2022), and others. In recent years, studies have indicated that using 3D printing bioink to treat bone tissue defects has good prospect, however bibliometric evaluation of this valuable field is currently lacking. To fill this gap, this study aims to perform bibliometric evaluation of the literature associated with the application of 3D printing bioink in bone tissue engineering from the past 13 years (2010–2022). We will conduct a systematic review of the current research situation of the main researchers, periodicals and institutions for the field and predict coming research trends and future improvement prospects in this field (Figure 1).

## 2 Materials and methods

### 2.1 Search strategy

**FIGURE 2:**
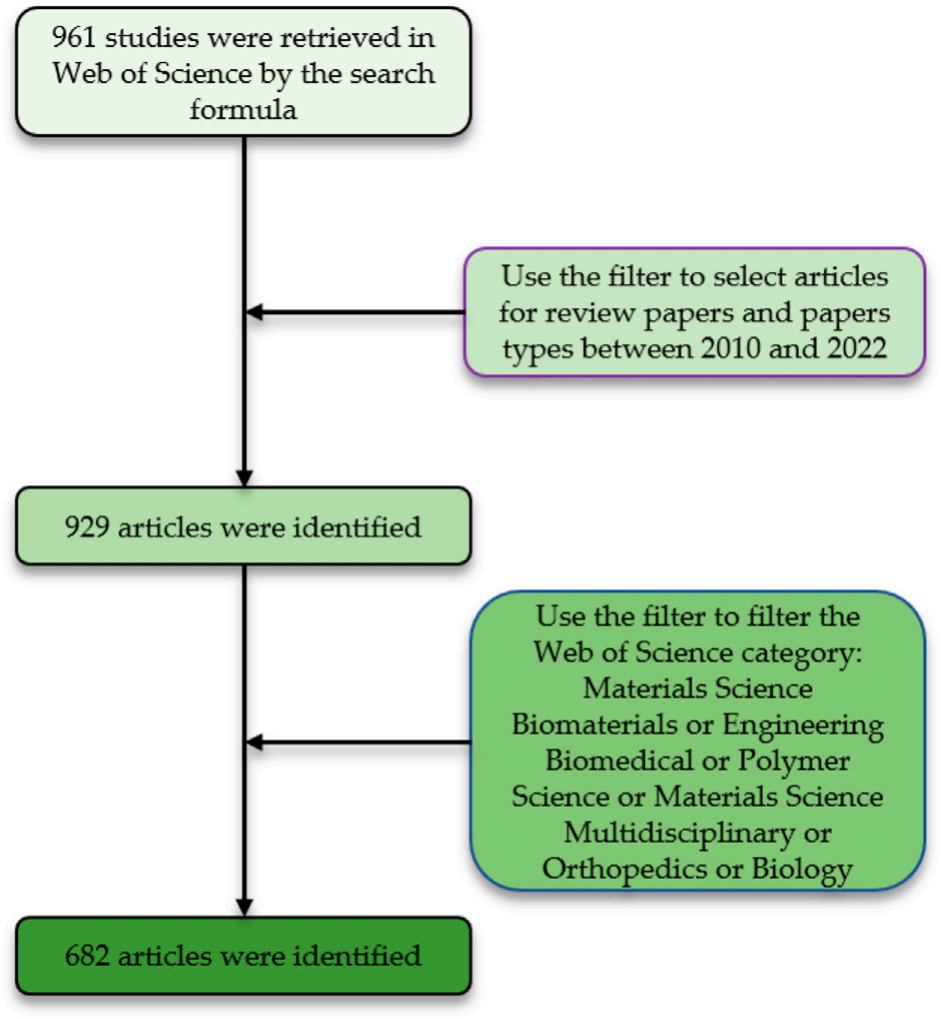
Flow chart of the literature screening process.

This paper focuses on 3D printing bioink in bone tissue engineering based on analysis of existing literature in the Web of Science (WOS) database. This database was chosen because it covers all areas related to medicine and is one of the most authoritative academic databases (Marín-Marín et al., 2021). The author systematically searched the citation index (SCI-EXPANDED) of the Web of Science core collection for articles published between 1 January 2010 and 31 December 2022 on 8 January 2023. To avoid bias, the document download process was completed within 1 day (8 January 2023). On the foundation of preceding studies, this study set the search strategy as the following: TS=((“hydrogel” OR “bioink” OR “ink” OR “biological ink”) AND (”3D printing” OR 3D) AND bone tissue engineering). A total of 961 documents were retrieved, and the author collated and identified the retrieved documents and further verified the article categories. 682 papers and review papers were ultimately included, and Figure 2 indicates the flow chart of literature inclusion.

### 2.2 Data analysis

Retrieved articles were screened by document type, publication year, and Web of Science category. 682 documents were exported as plain text files then imported into the VOSviewer (1.6.15.0) and CiteSpace (6.1.6.0) software, and bibliometric analysis was performed using the bibliometrix package (4.0.1) of R software (4.2.2).

The VOSviewer (1.6.15.0) bibliometric analysis software program analyzes underlying knowledge contained in scientific literature and visualizes the collected data (Xia et al., 2021). It can extract and analyze key information from many documents and is often used to establish co-citation and symbiotic networks (Vittori et al., 2022). The purpose of VOSviewer (1.6.15.0) used in this study is to analyze inter-country relations and research hotspots. For the analysis of inter-country relations, the type of analysis was chosen as co-authorship and the unit of analysis as countries. And to analyze research hotspots, the type of analysis was selected as co-occurrence, and the unit of analysis was selected as keywords plus. The counting method for all analyses is full counting. CiteSpace (6.1.6.0) is a statistical analysis tool based on the Java environment developed by Professor Chen Chaomei, which can be used for bibliometric analysis and visualization. It can analyze literature in a specific field and discover improvement trends of related disciplines (Yao et al., 2020). The CiteSpace (6.1.6.0) is used to analyze burst keywords and the trend of discipline evolution. In time slicing, follow-up analyzes were performed with years per slice of 2 years. Use the burstness detection option to detect top 16 keywords with the strongest citation bursts. At the same time, select the overlay maps function to analyze the trend of discipline evolution, select “Label Top N Journals” and “z Scores”, and then analyze the journal biplot overlay. R software is a language and environment widely used in the field of information statistics. In this study, the Bibliometrix package (4.0.1) in R (4.2.2) was used to analyze data and perform basic bibliometric analysis. Most local cited sources were extracted in the source option during the analysis. The information extracted by the authors option includes most relevant affiliations and corresponding author’s country. Meanwhile analyze the most local cited documents, most frequent words and the word cloud in the documents option. In the conceptual structure part, the thematic map and factorial analysis are selected for elaboration, and in the social structure section, the collaboration world map is analyzed.

Analysis of the 682 documents screened by the above software and programs provided results such as the most locally cited documents, most locally cited sources, most relevant words, country collaboration maps, factorial analysis, and thematic maps.

## 3 Bibliometric analysis and findings

### 3.1 Analysis of most localyl cited documents and sources in the field

#### 3.1.1 Most locally cited sources

**FIGURE 3:**
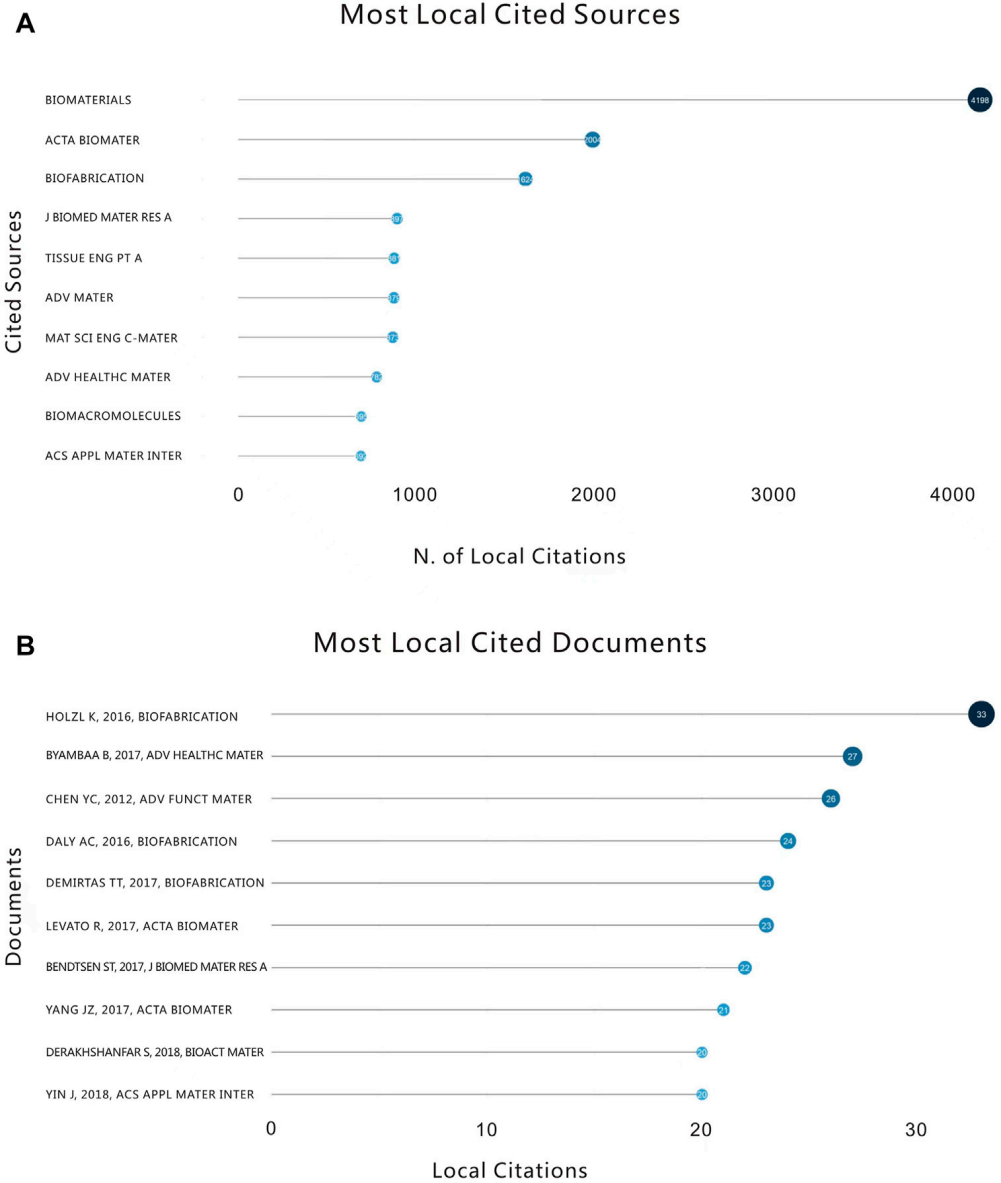
**(A)** The number of citations and the top ten highly cited journals in this field from 2010 to 2022. **(B)** The number of citations of highly cited documents in this field and the top ten articles from 2010 to 2022.

The 425 most locally cited journals in the field corresponding to the 682 included documents were analyzed, and the journals were ranked according to the number of articles. The top ten journals by number of articles are shown in Figure 3A, and it can be seen that the Biomaterials journal ranks first with 4198. Acta Biomater (2004) and Biofabrication (1624) ranked second and third, respectively. However, it is notable that the total number of articles in the second and third journals combined does not exceed the number of articles in Biomaterials. This indicates that Biomaterials has an authoritative super-status in the field of 3D printing bioink and that it has an essential guiding function in research in this field.

#### 3.1.2 Most locally cited documents

We next analyzed the most locally cited documents in this field by ranking the articles according to the number of citations in this field (Figure 3B). The article “Bioink properties before, during and after 3D bioprinting” published by Hölzl, K et al. in the Biofabrication Journal in 2016 was cited 33 times in this research field, with a total of 528 citations, ranking it first in number of citations. This article discusses the characteristics of related bioinks by taking the more common bioprinting methods (extrusion bioprinting, orifice-free bioprinting and inkjet bioprinting) as examples. Meanwhile, it additionally predicts the performance of hydrogels containing living cells (Hölzl et al., 2016). Displaying the most locally cited sources in the field can help researchers who dabble in the field for the first time to select optimal articles for selective reading.

### 3.2 Analysis of affiliations and countries

#### 3.2.1 Most relevant affiliation

**FIGURE 4:**
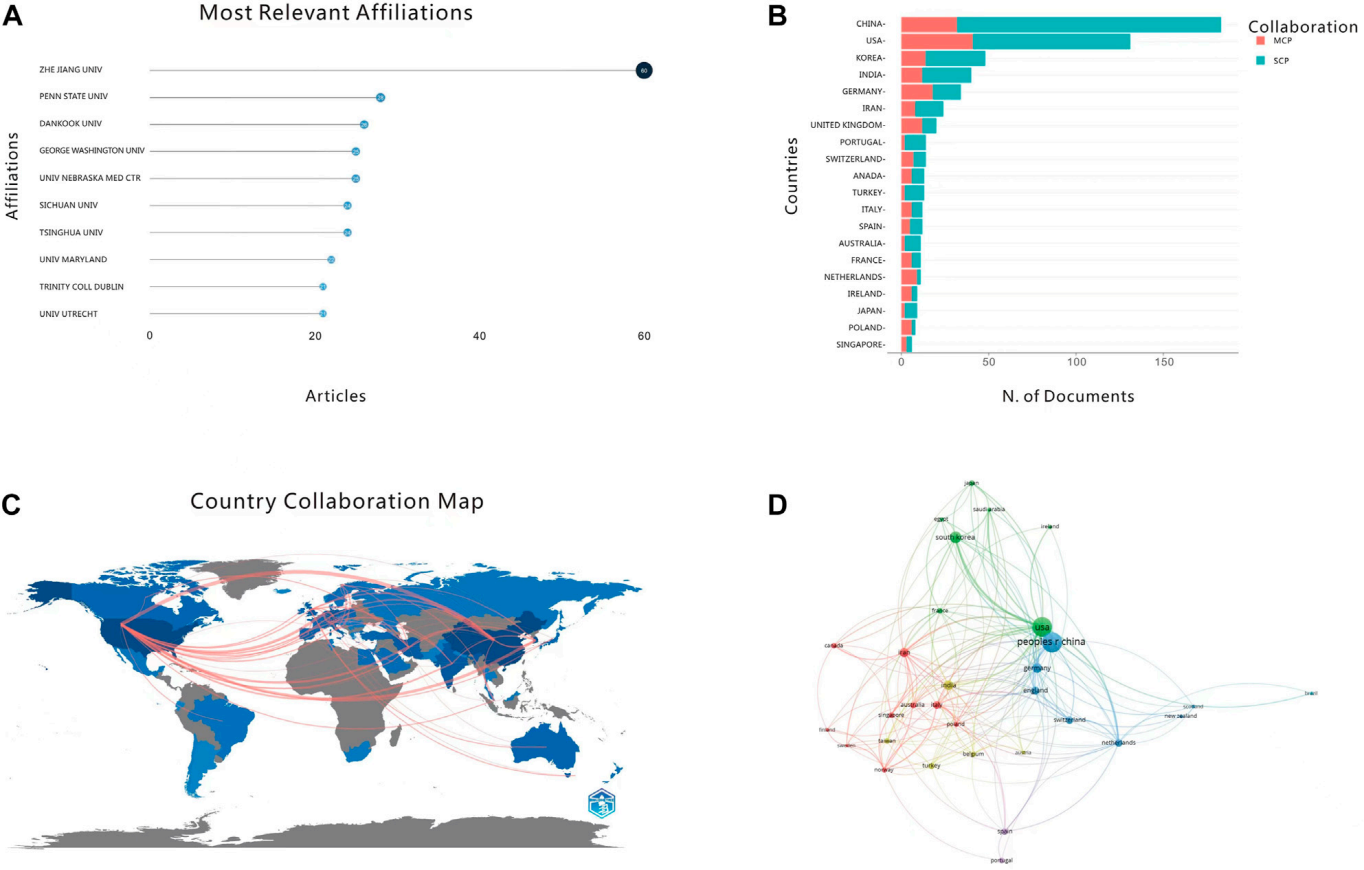
**(A)** Top 10 most relevant affiliations. **(B)** Top 20 most productive countries. **(C)** Distribution of publications by countries and regions. **(D)** National cooperation map.

Analyzing 948 research institutions (universities), Figure 4A reveals the ten major research institutions that published the most articles in this field. Zhejiang University in the People’s Republic of China (60) had the most relevant research publications on the application of 3D printing bioink in bone tissue engineering, followed by the University of Pennsylvania in the United States (28) and Dankook University in South Korea (26). This indicates that Zhejiang University has high research achievements in the field of 3D printing bioink. The top ten major research institutions in this field are affiliated with the United States 4), China 3), South Korea 1), Ireland 1) and the Netherlands 1), which indicates that Asia, North America and Europe may have high research standards.

#### 3.2.2 Corresponding author’s country

An analysis of the nationality of the corresponding authors demonstrates that China dominates this field of research (Figure 4B). Collaborations between scientists within one country are represented by Single country publications (SCP), while the worldwide partnerships are represented by multiple country publications (MCP). Our search determined that China has the largest number of corresponding authors within this field, with the United States ranks second. The Chinese SCP value far exceeds that of other countries, while the MCP value of the United States exceeds China’s. This may infer that China has established capabilities to complete research projects in this field relatively independently, while the United States has stronger international cooperation capabilities. Analysis of the corresponding author’s country also confirms results quantifying main research institutions from another perspective, and the number of corresponding authors in countries with a large number of main research institutions is more than that of other countries.

#### 3.2.3 Analysis of the cooperative relationship between countries

Based on the above two analyses, international collaborations were filtered and visualized by number of publications to construct a collaboration network based on the number of publications and relationships between countries (Figures 4C, D). There are notably differing degrees of cooperation among different countries. In Figure 4C, the shades of different countries on the map represent the number of articles from that country. The thickness of connecting lines indicates the cooperative relationship between two countries (regions), with wider connecting lines denoting stronger cooperative relationships. Thinner lines indicate that, although there is cooperation between countries, the cooperative relationship is not very close. For example, China shows close cooperation with the United States, the United Kingdom, Germany, and South Korea, while the United States shows productive cooperation with Iran, South Korea, and the Netherlands.

### 3.3 Analysis of keywords

**FIGURE 5:**
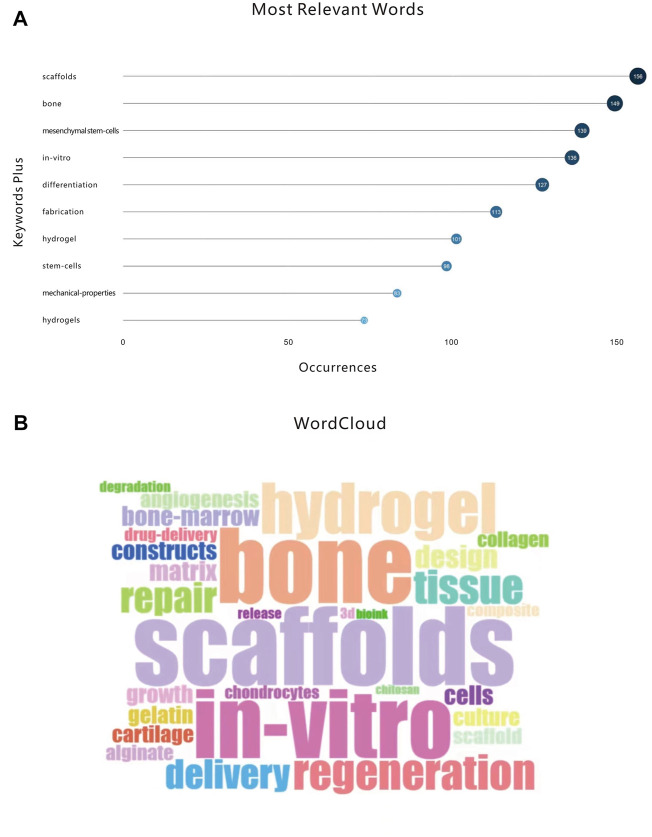
**(A)** Top 10 most relevant words. **(B)** The WordCloud.

We next analyzed the most frequent words across literature from the field to indentify commonly used phrases and to measure their frequency (Figure 5A). After sorting out the top ten high-frequency words, “scaffolds (156)” appears the most. By searching the WordCloud Map “number of words>50” (Figure 5B), we revealed that the most frequently occurring words are consistent with the results obtained for the word cloud. These two analyses uncovered the most frequently occurring words in this field, revealing which topics receive focus in the published research.

### 3.4 Analysis of research hotspots

**FIGURE 6:**
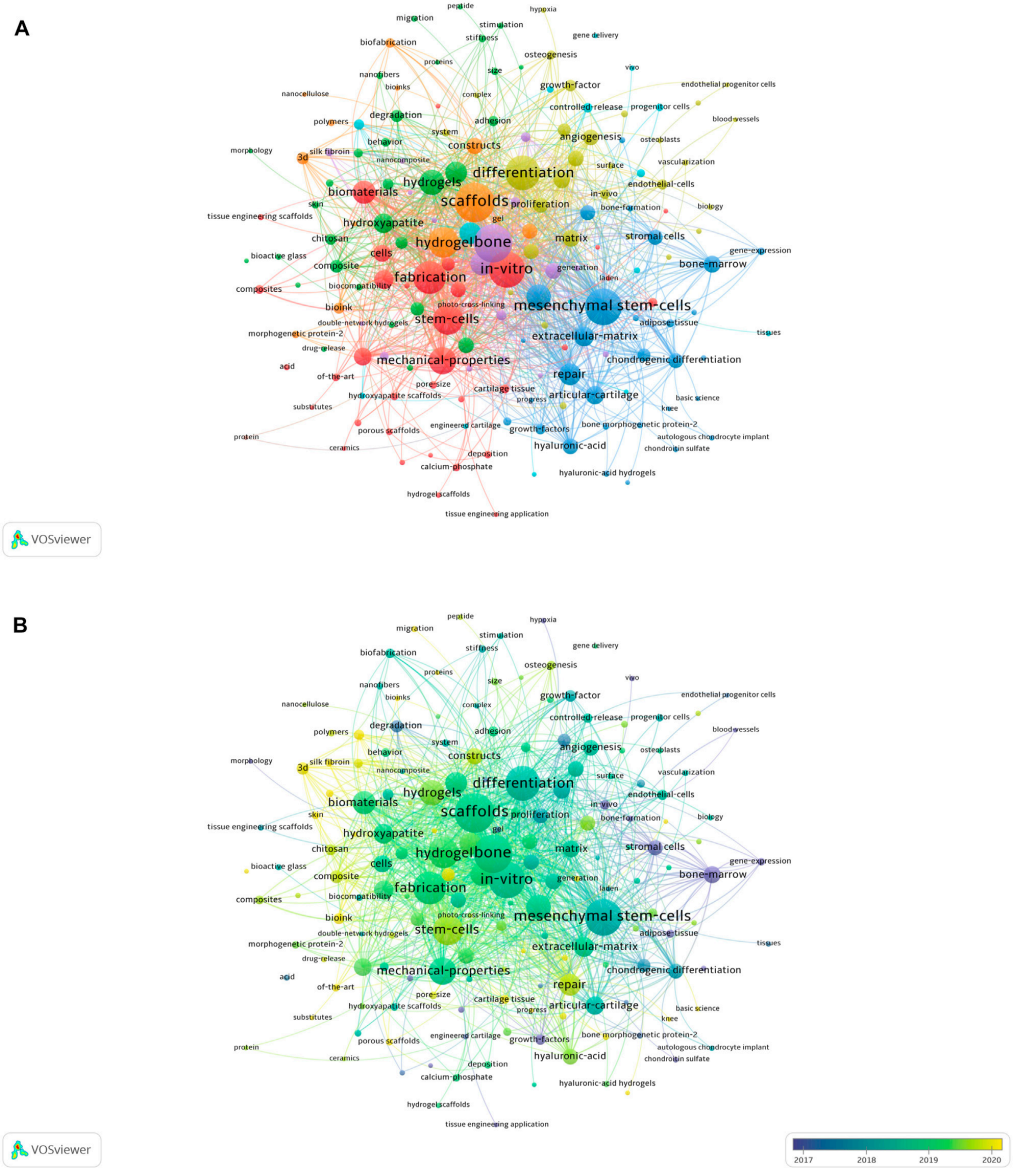
**(A)** Keywords related to the application of 3D printing bioink in bone tissue engineering are divided into 5 clusters according to different colors. Cluster 1: red; Cluster 2: green; Cluster 3: blue; Cluster 4: yellow; Cluster 5: purple. The dimension of the node shows the frequency of prevalence of the word. **(B)** Visualizing keywords based on average publication year. Purple keywords appear earlier than yellow keywords.

VOSviewer was used to analyze keywords and subject headings extracted from the titles and abstracts of 682 papers (Figure 6). As seen in Figure 6A, cluster 1 is mainly discussion of *in-vitro* research on 3D printing bioink in bone tissue engineering. The main keyword of cluster 2 is hydrogels, and this discusses the hydrogel materials formed by mixing materials including hydroxyapatite. Cluster 3 largely discusses the relationship among mesenchymal stem cells, stromal cells and extracellular matrix in bone tissue engineering. Cluster 4 focuses on the differentiation and proliferation of tissue structures such as collagen and angiogenesis in bone tissue engineering. Cluster 5 generally discusses the formation of bone and cartilage tissue in bone tissue engineering. The keywords with the highest frequency are “*in-vitro*”, “hydrogels”, “mesenchymal stem-cells”, “differentiation” and “bone”, indicating that research on 3D printing bioink in bone tissue engineering mainly focuses on material formation and tissue differentiation.

We further used VOSviewer to color-code all keywords according to average publication year (Figure 6B). This analysis can visually distinguish when keywords occur, revealing trends, evolutionary processes, and emerging areas of research.

### 3.5 Thematic map

**FIGURE 7:**
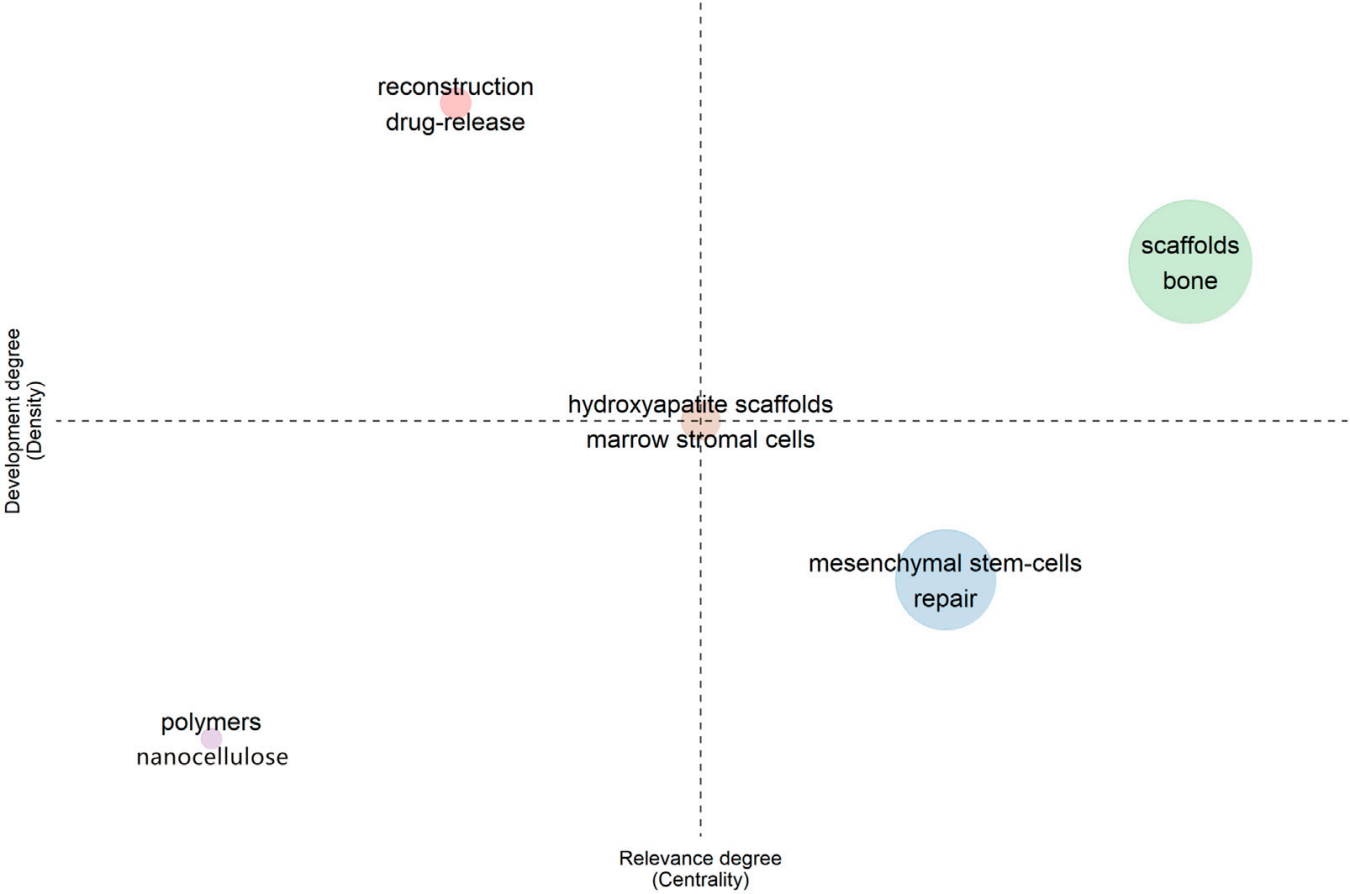
Strategic theme map.

Thematic Maps help identify conceptual evolution across various topics by creating a thematic cluster from co-word analysis. The program identifies clusters based on density and centrality then names them according to the most frequently occurring keywords in the cluster (Figure 7). Density indicates how often a cluster is internally linked, and centrality measures how often a cluster (topic) is linked to other topics. High density indicates greater development within a topic, while high centrality means more connections to other topics, or a particular topic being central to a greater field of study. In the thematic map, each quadrant has an intuitive meaning. Density is represented on the vertical axis (*Y*-axis), while centrality is represented on the horizontal axis (*Y*-axis). The quadrant distribution is as follows:

The first quadrant (upper right) represents the topic of scaffold osteogenesis, a quadrant that demonstrates the well-established conceptual foundation and robustness of this field of study.

The second quadrant (upper left) is drug release and reconstruction. This quadrant represents a well-developed but isolated theme.

The third quadrant (lower left) is devoted to polymers and nanocellulose, and this quadrant represents rising or disappearing themes.

The fourth quadrant (lower right) is repair of mesenchymal stem cells. These topics have not been well researched and developed, but they are often present in the research scope and may be a hot research field in the future.

It is worth mentioning that “hydroxyapatite scaffolds” and “marrow stromal cells” locate in the center of the four quadrants. This is an interesting phenomenon, probably indicating that these studies have been developed to a certain extent, but that the relationship between disciplines is not close enough. As such, these topics show research value but do not often appear in the greater existing literature.

### 3.6 The trend of discipline evolution

**FIGURE 8:**
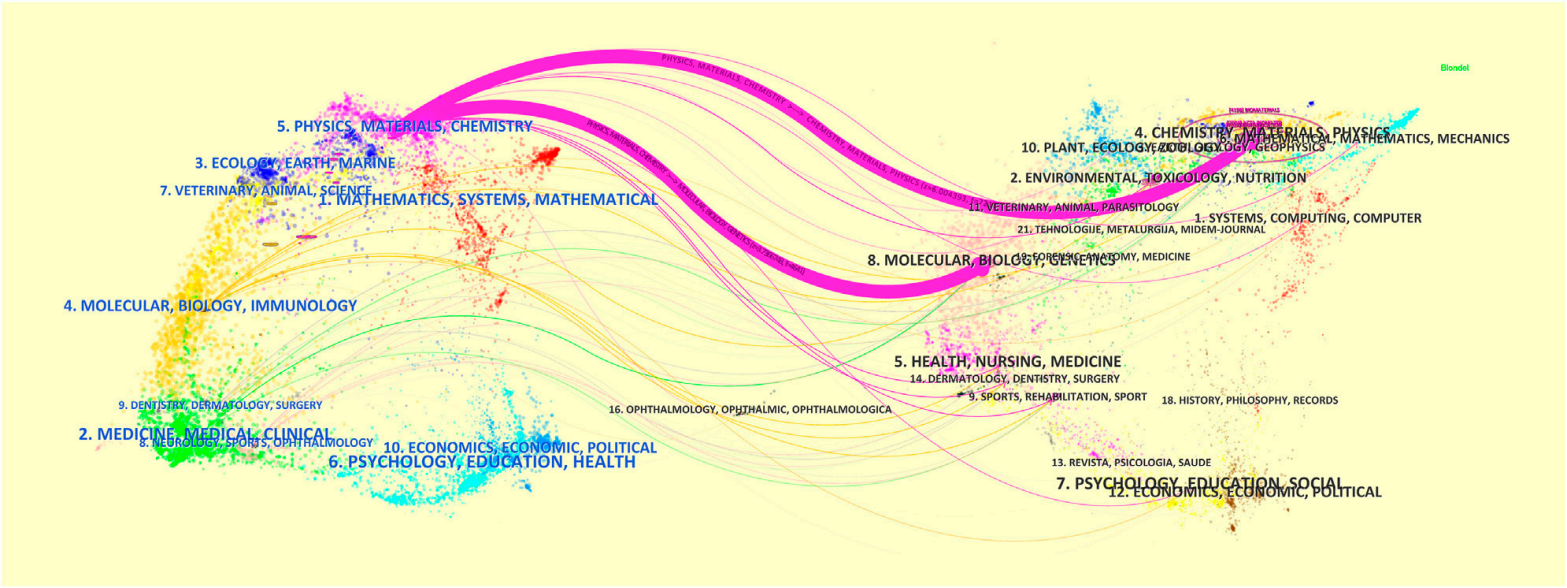
Double-journal overlay of research on the application of 3D printing bioink in bone tissue engineering.

The double-picture superposition of periodicals shows the citation relationship between journals and co-cited periodicals, with citation periodicals on the left and cited periodicals on the right. As shown in Figure 8, the essential reference paths are the two purple paths. The upper purple path represents that literature published in journals of CHEMISTRY, MATERIALS, and PHYSICS is mainly cited by other studies published within journals of PHYSICS, MATERIALS, and CHEMISTRY. The lower purple path represents that literature published in MOLECULAR, BIOLOGY, and GENETICS journals is mainly cited by papers within PHYSICS, MATERIALS, and CHEMISTRY journals.

### 3.7 Factorial analysis and burst keywords

#### 3.7.1 Factorial analysis

**FIGURE 9:**
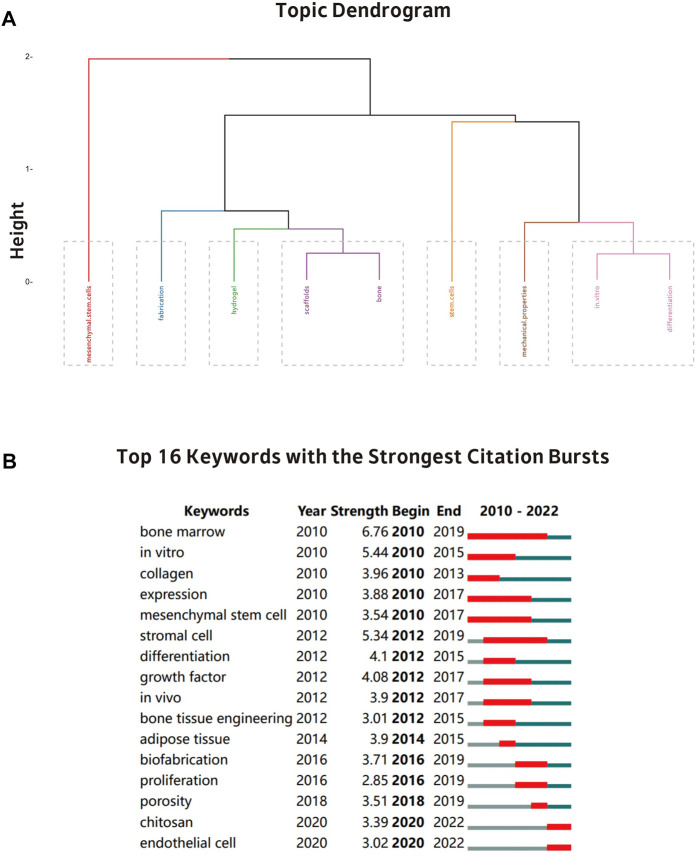
**(A)** A dendrogram showing the broadest evolution of 3D printing bioink in the discipline of bone tissue engineering. **(B)** A visualization of the top 16 citation bursts keywords produced by CiteSpace.

A review of selected literature resulted in some general observations. First, clustering via factorial analysis resulted in seven exclusive clusters (Figure 9A): mesenchymal stem cells; fabrication; hydrogel; scaffolds; bone; stem cells; mechanical properties; *in vitro*; differentiation. These classes are not mutually exclusive and there is overlap between different categories.

#### 3.7.2 Analysis of keywords with citation burst

Figure 9B shows the Top16 keywords with the shortest citation burst time of 1 year. Keywords such as “bone marrow” (2010–2019), “expression” (2010–2017), “mesenchymal stem cell” (2010–2017), and “stromal cell” (2012–2019) received the longest attention in the past 13 years. In contrast, keywords such as “chitosan” (2020–2022) and “endothelial cell” (2020–2022) appear for a short time and have been used more frequently in recent years, indicating that these keywords have recently attracted attention and may be potential upcoming hotspots for future research.

## 4 Discussion

### 4.1 Research status

Through discussing the research trends of the seven categories of factorial analysis, the multivariate relationships between various field components can be revealed, intuitively highlighting the current status of research in this area. As one of the most transplanted tissues in the world bone is often used to treat patients with congenital bone defects, trauma, infection, or tumor resection (Graham et al., 2010). The main cellular components of bone are osteoblasts that form bone and osteoclasts that resorb bone. The activity-regulated balance of both phenomena is responsible for bone formation and tissue repair (Salhotra et al., 2020). The main functional cells responsible for bone tissue formation are osteoblasts, which perform the synthesis, secretion, and mineralization of bone matrix. (Kim et al., 2020). Osteoblasts can be derived from stem cells, making stem cells key to the future of bone tissue engineering and regeneration. Common stem cell populations in this field include bone marrow mesenchymal stem cells, adipose tissue-derived mesenchymal stem cells, dental pulp stem cells, human umbilical vein endothelial cells, periosteum-derived stem cells, and trabecular bone progenitor cells (Szpalski et al., 2012). Mesenchymal stem cells have been widely studied in bone tissue engineering and regenerative medicine because of their extensive proliferation ability and pluripotency (Naji et al., 2019; Tsiapalis and O'Driscoll, 2020). Bone marrow mesenchymal stem cells are the most studied cell type to date in this field. Bone tissue engineering scaffolds containing bone marrow mesenchymal stem cells have the required mechanical strength and also provide excellent cell delivery ability, with evenly distributed cells improving bone formation ability *in vivo* (He et al., 2012). To promote bone tissue regeneration, the scaffold structure needs to have sufficient mechanical strength and be able to bear the pressure of soft and hard tissues (Wang et al., 2019). Researchers have been looking for materials with improved bone tissue scaffolding properties, and alginate is one of the most common materials used to make bioinks (Mrudulakumari Vasudevan et al., 2021). Due to its chemical inertness and superior gel properties, alginate can form bone tissue engineering scaffolds with more stability and optimized mechanical properties (He et al., 2022). Common types of scaffolds for bone tissue engineering include films, hydrogels, fibers, and nanospheres (Chen et al., 2010). In recent years, hydrogels have attracted interest in the field of bone tissue engineering due to their unique physical properties including swelling and diffusion capabilities (Hasani-Sadrabadi et al., 2020). The elasticity, biodegradability, and other characteristics of hydrogels mainly depend on the composition of the hydrogel. Due to differences in composition, hydrogels are mainly divided into solid (Coyne et al., 2020), semi-solid, (Hamd-Ghadareh et al., 2022), and liquid hydrogels (Wang et al., 2022) according to their physical properties. After 3D printing of bioink, further *in vitro* research is required. Common *in vitro* studies include cell culture and maintenance, cell viability assays, live-dead cell experiments, mineralization studies, and more. Following a number of quality tests, the hydrogels can be implanted in the living body. *In vivo* experiments are then conducted to observe its osteogenic ability (Huebsch, 2019).

The results of the strongest citation burst keywords analysis show that chitosan and endothelial cells have received high attention in recent years. Chitosan is the second most abundant natural polymer on earth and has wide applications in the field of biomaterials (Chang et al., 2022). Due to its unique dynamic reversibility and excellent biological properties, chitosan supramolecular hydrogel is considered an ideal material for 3D bioprinting bioink in bone tissue engineering (Xu J. et al., 2022). The vascular endothelial network also plays a crucial role in bone tissue formation and regeneration. Endothelial cells assist in vascularization while also influencing bone physiology through cell-contact-dependent mechanisms (Mutschall et al., 2020). For this reason, researchers are increasingly focusing on endothelial cells, and with improved bioink materials, 3D printing can promote effective vascularization processes (Qiu et al., 2020).

### 4.2 Research hotspots and prospects

Bone and cartilage regeneration is one of the hottest areas of research with 3D printed bioinks. Compared with traditional techniques, the application of 3D printing bioink in bone tissue engineering can more accurately control the structure and mechanical properties of artificial scaffolds (Li et al., 2016). At this stage and in the future, research in this field will likely be devoted to developing bioinks with different properties. By adding modified materials, bioinks could have better printability, stability, mechanical load capacity, and osteogenesis potential (Midha et al., 2019). The emerging research hotspot of microsphere structure can provide biomimetic structure and biological performance for cell growth and give scaffolds a more stable porous structure and a higher specific surface area (He et al., 2020). Since microspheres can provide a porous network, their pores can be loaded with growth factors, drugs, or nanophase materials (Gupta et al., 2017). Presently, the exploration of hydroxyapatite microspheres has become popular. Hydroxyapatite microspheres can be prepared in several ways including template, hydrothermal, spray drying, microemulsion, and precipitation methods. Using polyvinyl alcohol-modified hydroxyapatite microspheres as a bioink, 3D scaffolds with strong mechanical properties and inorganic components similar to natural bone can be successfully printed (Wei et al., 2022). Metal ions can also modify hydroxyapatite microspheres, such as carboxylated chitosan/silver-hydroxyapatite hybrid microspheres. Because of the synergistic effect of silver, they can also exhibit excellent antibacterial activity against *Staphylococcus aureus*, allowing them to be used as an anti-infection bone substitute material (Shen et al., 2017). In addition to hydroxyapatite microspheres, materials such as alginate (Xu M. et al., 2022) and *ε*-polylysine (Ge L. et al., 2022) have also been studied to prepare microspheres. In the future, preparing materials into microspheres and adding them to bioinks to improve properties will become a research hotspot in 3D printing bioink for bone tissue engineering. In recent years, the addition of bioactive glass to bioinks has also attracted widespread attention from scientists (Zeimaran et al., 2021). After bioactive glass is implanted into the human body, bioactive hydroxyapatite can form over time, promoting bone tissue regeneration. For example, copper-doped mesoporous bioactive glass can improve the printability and printing accuracy of bioinks and give the bioinks improved osteogenesis and angiogenesis abilities (Zhu et al., 2022). At the same time, with the deepening of the research, the researchers found that graphene oxide (GO) can induce the differentiation of mesenchymal stem cells into osteoblasts, which is helpful for the recovery of defective bone tissue. As popular materials that can improve the physical and chemical properties of scaffolds, metal and non-metal materials can be combined with GO to exert a more excellent osteogenic effect. For example, adding Fe_3_O_4_ to the scaffold material can greatly improve the mechanical strength of the porous scaffold and improve the antibacterial performance through the interaction with GO (Khan et al., 2022c). The combined effect of zinc and GO can improve cell viability and cell proliferation, thus becoming a potential biomaterial for bone tissue engineering (Khan et al., 2022a). The addition of SiO_2_ can make the scaffold material containing GO have more excellent cell adhesion ability, cell viability and proliferation ability (Khan et al., 2022b). It can be seen that the combination of different metals and non-metals with GO will have different effects, and the bioink material belonging to the patient can be customized according to the specific demands of the patient. To sum up, whether it is microsphere technology, the combination of GO and metal or non-metallic materials, or adding modified materials such as bioactive glass to improve the performance of bioink, current research is in the early phases of exploration. Future research will focus on improving bioinks to make them more suitable for bone tissue engineering.

Although research into 3D printing bioink for bone tissue engineering has grown in the past decade, its current limitations cannot be ignored. Despite the high precision and repeatability of 3D printing, the main technical challenge remains to explore bioinks with good biocompatibility and mechanical strength (Zhang et al., 2021). Scientists are also working on exploring how to ensure complete preservation and effective implantation of bioink during the 3D printing process, as well as how to ensure good integration of new bone grown in bone defects with the original bone tissue. Personalized 3D printing technology is also a relatively new technology (Kumar Gupta et al., 2022), therefore, there are still certain obstacles in the supervision of printing products. Relevant laws and regulations need to be established and improved to ensure the sustainable development of 3D printing technology (Prabhakaran et al., 2022). These limitations may also become a research hotspot in the future of 3D printing bioink in the field of bone tissue engineering.

## 5 Conclusion

Bone tissue defect is a common clinical disease. The long treatment period of traditional treatment methods will cause huge psychological pressure and economic burden to patients. Therefore, the treatment of bone defects remains one of the major clinical and scientific challenges. Surgical treatment is a traditional method for treating bone defects, but problems such as long treatment period and difficult operation have always plagued doctors and patients. In recent years, the rapid development of bone tissue engineering has provided new therapeutic ideas for bone repair. Scaffold materials with bioactivity and biodegradability gradually come into people’s sight. Bioactivity promotes cell proliferation, while biodegradability can avoid secondary surgery trauma to patients. By combining 3D printing technology based on computer design and manipulation, doctors can personalize design the stent shape according to the patient’s actual situation based on the shape of the bone defect. While reducing the economic and psychological pressure of patients, formulate personalized treatment plans that belong to patients to the greatest extent.

Research on the application of 3D printing bioink for bone tissue engineering has attracted growing attention in the past decades. This study includes 682 papers retrieved and screened from the Web of Science database, and analyzed using the bibliometrix R package and VOSviewer and CiteSpace software. The study found that China ranks first in the number of publications in this field, while the SCP of the United States surpasses China and ranks first. Correspondingly, major research institutions are also concentrated in the United States and China. This study identifies key researchers and institutions globally involved in this field. The journal Biomaterials is the most prolific journal in the field, and Hölzl’s article published in the journal Biofabrication in 2016 is the most cited in the field. The term “scaffolds” appears most frequently across the field, indicating that it has received extensive research and attention. Chitosan and endothelial cells may additionally emerge as the focal point of future research. These findings provide new researchers and policymakers with a comprehensive perspective on the broader prospects of this research field. It is undeniable that this study has certain limitations, while innovatively using bibliometric methods to analyze the application of 3D printing bioinks in bone tissue engineering. This study is based on the analysis of previous studies, and there will be a certain lag. Works including unpublished articles, topics under research, and non-English literature are not included in the statistics. It is worth noting that in addition to basic research, attention should also be paid to the results of translational research. The ability to shorten the time of bone tissue reconstruction and reduce economic costs will promote the transformation of 3D printing bioink from theoretical experiments to clinical practice in the field of bone tissue engineering.

## Data Availability

The original contributions presented in the study are included in the article/Supplementary material, further inquiries can be directed to the corresponding authors.
